# Hypertriglyceridemia Is Independently Associated with Renal, but Not Retinal Complications in Subjects with Type 2 Diabetes: A Cross-Sectional Analysis of the Renal Insufficiency And Cardiovascular Events (RIACE) Italian Multicenter Study

**DOI:** 10.1371/journal.pone.0125512

**Published:** 2015-05-05

**Authors:** Giuseppe Penno, Anna Solini, Giacomo Zoppini, Cecilia Fondelli, Roberto Trevisan, Monica Vedovato, Gabriella Gruden, Olga Lamacchia, Antonio E. Pontiroli, Maura Arosio, Emanuela Orsi, Giuseppe Pugliese, for the Renal Insufficiency And Cardiovascular Events (RIACE) Study Group

**Affiliations:** 1 Department of Clinical and Experimental Medicine, University of Pisa, Pisa, Italy; 2 Division of Endocrinology and Metabolic Diseases, University of Verona, Verona, Italy; 3 Diabetes Unit, University of Siena, Siena, Italy; 4 Endocrinology and Diabetes Unit, Azienda Ospedaliera Papa Giovanni XXIII, Bergamo, Italy; 5 Department of Clinical and Experimental Medicine, University of Padua, Padua, Italy; 6 Department of Internal Medicine, University of Turin, Turin, Italy; 7 Department of Medical Sciences, University of Foggia, Foggia, Italy; 8 Department of Medicine, Surgery and Odontoiatrics, San Paolo Hospital, University of Milan, Milan, Italy; 9 Endocrinology Unit, San Giuseppe Hospital, University of Milan, Milan, Italy; 10 Diabetes Unit, IRCCS “Cà Granda—Ospedale Maggiore Policlinico” Foundation, Milan, Italy; 11 Department of Clinical and Molecular Medicine, “La Sapienza” University, Rome, Italy; Weill Cornell Medical College in Qatar, QATAR

## Abstract

**Objective:**

Atherogenic dyslipidemia seems to play a major role in microvascular complications and in residual microvascular risk after statin therapy, which reduces triglycerides up to 40%. We assessed whether raised TG levels are associated with an increased burden from microvascular complications in patients with type 2 diabetes.

**Methods:**

Subjects from the Renal Insufficiency And Cardiovascular Events (RIACE) Italian Multicentre Study (n=15,773) were divided in 4 groups depending on whether they had plasma triglycerides below (NTG, 67.8%) or above (HTG, 32.2%) 1.7 mmol/L and were (42.4%) or not on (57.6%) statin therapy. Estimated GFR (eGFR) was calculated from serum creatinine, albuminuria was measured by immunonephelometry or immunoturbidimetry, and retinopathy was evaluated by fundus examination.

**Results:**

HTG subjects, either with or without statin, had higher prevalence of albuminuria, reduced eGFR and chronic kidney disease (CKD), especially the albuminuric forms, but not of retinopathy, than NTG subjects. In contrast, cardiovascular disease and advanced DR were more prevalent in subjects on statin than in those not, independently of triglyceride levels. Logistic regression analysis confirmed that HTG, without or with statin, was independently associated with micro and macroalbuminuria, mildly to severely reduced eGFR, and all CKD phenotypes, but not with retinopathy. The adjusted odd ratios for CKD increased linearly for every 0.26 mmol/L increase (approximately one decile) in triglyceride levels. The increase was higher with increasing severity of albuminuria, eGFR loss and CKD phenotype as well as in subjects receiving than in those not receiving statin treatment.

**Conclusions:**

Triglycerides are associated with CKD, but not retinopathy in subjects with type 2 diabetes, independently of statin treatment. These data point to a possible role of hypertriglyceridemia in the development of CKD, though it remains to be demonstrated that diabetic individuals might benefit from triglyceride reduction with statins and eventually with combination therapy with fibrates.

**Trial Registration:**

www.ClinicalTrials.gov NCT00715481

## Introduction

Elevated LDL cholesterol is a major risk factor for cardiovascular disease (CVD), as indicated by the linear relationship between reduction in LDL cholesterol levels and decrease in CVD event rates in trials with statins, which inhibit the rate limiting enzyme of cholesterol synthesis [1]. A recent meta-analysis has shown that statins are as effective in diabetic as in nondiabetic individuals [2]. Indeed, due to the higher CVD risk associated with diabetes, the absolute reduction in CVD risk is higher in diabetic than in nondiabetic patients, though event rates in diabetic subjects on statin therapy exceed those of placebo-treated individuals without diabetes [3]. However, significant residual CVD risk remains after statin treatment, even with intensive regimens targeting an LDL cholesterol level below 70 mg/dl [4]. This residual risk has been attributed, at least in part, to atherogenic dyslipidaemia, since it is higher in subjects with raised triglyceride levels [5] and/or low concentrations of HDL cholesterol [6], who benefit from specific treatments such as fibrates, either alone [7] or in combination with statins [8]. Moreover, levels of triglycerides [9] and HDL cholesterol [10] are positively and negatively associated, respectively, with CVD risk, independently of LDL cholesterol levels.

Recently, a growing body of evidence has suggested that atherogenic dyslipidaemia might play a major role also in microvascular complications and contribute to residual microvascular risk after intensive multifactorial treatment including statin therapy [11]. In fact, the role of LDL cholesterol in microvascular disease is less clear, as suggested by statin trials showing no effect on diabetic retinopathy (DR) [12] and modest, if any, impact on chronic kidney disease (CKD) [13,14]. Conversely, most epidemiological studies found an association of triglycerides and, less consistently, HDL-cholesterol with diabetic nephropathy [15,16], whereas contrasting data exist on the relation between levels of these lipid subfractions and DR [17,18]. In addition, fenofibrate, either alone or combined with statins, reduced decline in renal function, albuminuria, and requirement for laser treatment [19–21], though these effects were not related to improvements in triglycerides or HDL-cholesterol levels.

Despite evidence for a major role of atherogenic dyslipidaemia in both macro and microvascular complications and the high prevalence of this abnormal lipid pattern, especially in subjects with type 2 diabetes [22], statin monotherapy is still the most used lipid-lowering treatment in these individuals [23]. However, statins reduce triglycerides by up to 40%, depending on baseline levels, whereas their effect on HDL cholesterol is less pronounced [24].

This analysis was aimed at assessing whether hypertrygliceridemia, independently of statin treatment, is associated with an increased burden from microvascular complications in subjects with type 2 diabetes.

## Subjects and Methods

### Patients

In this cross-sectional analysis, we used the data collected at the baseline visit for the Renal Insufficiency And Cardiovascular Events (RIACE) Italian Multicentre Study using standardized protocols across study centers [25]. The RIACE Study (registered with ClinicalTrials.gov, NCT00715481; URL http://clinicaltrials.gov/ct2/show/NCT00715481) is an observational, prospective cohort study on the impact of estimated glomerular filtration rate (eGFR) on morbidity and mortality from CVD in subjects with type 2 diabetes. Data were collected in a single database using a dedicated computer software developed by Client-Server.net (Belluno, Italy). The study was conducted in accordance with the Declaration of Helsinki and the protocol was approved by the coordinating center’s Ethic Committee (Comitato Etico dell’Azienda Ospedaliera Sant’Andrea, Prot. n. 43/2006) and thereafter by the Ethics Committee of each center. Participants gave informed consent for participation in the RIACE study, as reported in electronic records. Consent was either written or verbal, depending on the specific recommendations of the Ethics Committees, some of which did not request to obtain written consent because (a) data were anonymized prior to access for this study; (b) only measures required for routine follow-up of diabetic patients were obtained; and (c) no blood or other biological sample was collected from participants for additional measurements.

The RIACE cohort consisted of 15,933 Caucasian patients with type 2 diabetes (defined by the American Diabetes Association criteria), attending consecutively 19 hospital-based Diabetes Clinics of the National Health Service throughout Italy (see S1 RIACE Investigators) in years 2007–2008. Exclusion criteria were dialysis or renal transplantation.

The quality and completeness of data were controlled and 160 patients were excluded due to missing or implausible values; data from the remaining 15,773 patients were subsequently analyzed.

### Measurements

All patients underwent a structured interview in order to collect the following information: age, smoking status, known diabetes onset and duration, current glucose-, blood pressure (BP)- and lipid-lowering therapy, with indication of the class of drug. Body weight and height were assessed and body mass index (BMI) was calculated as weight (kg) / height (m^2^), then BP was measured with a sphygmomanometer after a five min of rest with the patients seated with the arm at the heart level. Hypertension was defined by systolic BP ≥140 mmHg and/or diastolic BP ≥90 and/or anti-hypertensive treatment. Hemoglobin (Hb) A_1c_ was measured by high-performance liquid chromatography using DCCT-aligned methods; triglycerides, total and HDL cholesterol were determined by colorimetric enzymatic methods; LDL cholesterol was calculated by the Friedwald formula, i.e.: LDL-cholesterol (in mmol/L) = total cholesterol—[HDL-cholesterol + (triglycerides/2.17)].

The presence of CKD was assessed by measuring albuminuria and serum creatinine. As previously reported in detail [25,26], albumin excretion rate (AER) was obtained from timed (24 hour) urine collections or calculated from albumin/creatinine ratio in early-morning, first-voided urine samples, in the absence of symptoms and signs of urinary tract infection or other interfering clinical conditions. Albuminuria was measured in one-to-three fresh urine samples for each patient by immunonephelometry or immunoturbidimetry and, in case of multiple measurements, the geometric mean was used for analysis. In subjects with multiple measurements (4,062 with at least two and 2,310 with three values), concordance rate between the first value and the geometric mean was >90% for all classes of albuminuria [26]. Patients were then assigned to one of the following categories of albuminuria (mg/24 hours): normoalbuminuria (AER <30), microalbuminuria (AER 30–299), or macroalbuminuria (AER ±300). Serum (and urine) creatinine was measured by the modified Jaffe method. One to three measurements were obtained for each patients and eGFR was calculated by the four-variable Modification of Diet in Renal Disease (MDRD) Study equation [27], using the mean serum creatinine value in case of multiple measures, as reported in previous publications [25,26]. Patients were then assigned to one of the following categories of eGFR (mL/min/1.73 m^2^): 1 (±90); 2 (60–89); 3 (30–59); 4 (15–29); and 5 (<15). Finally, subjects were classified as having no CKD or CKD Stages 1–5, based on the presence or absence of micro or macroalbuminuria and the value of eGFR, according to the National Kidney Foundation’s Kidney Disease Outcomes Quality Initiative [28]. Patients assigned to CKD Stages (and GFR classes) 4 and 5 were pooled together. As previously reported [25], CKD patients were further classified as having one of the following CKD phenotypes: albuminuria alone (CKD Stages 1–2), reduced eGFR alone (CKD Stage ±3 without albuminuria), or both (CKD Stage ±3 with albuminuria).

In each centre, the presence of DR was assessed by an expert ophthalmologist with dilated fundoscopy. Patients were classified into the following categories: absent DR, mild, moderate or severe non-proliferative DR, proliferative DR, or maculopathy, according to the Global Diabetic Retinopathy Project Group [29]. Patients were classified based on the actual fundus appearance or the retinal disease condition which had eventually required a previous photocoagulation or surgical treatment. For further analysis, patients with non-proliferative DR of mild or moderate degree were classified as having non-advanced DR, whereas those with severe non-proliferative DR, proliferative DR, maculopathy, or blindness were grouped into the advanced, sight-threatening DR category. Subjects with maculopathy and non-advanced DR were classified as having maculopathy, whereas those with maculopathy and severe non-proliferative DR or proliferative DR were classified as having one of the latter conditions [30]. DR grade was assigned based on the worst eye.

Prevalent CVD was assessed from medical history by recording previous documented major acute CVD events, including myocardial infarction, stroke, foot ulcer or gangrene, amputation, coronary, carotid, and lower limb revascularization. CVD events were adjudicated based on hospital discharge records by an *ad hoc* committee in each center [31].

### Statistical analysis

Since statin treatment influences triglyceride levels, patients were divided in 4 groups depending on whether they (a) had plasma triglycerides concentrations below or above 1.70 mmol/L (normal triglycerides [NTG] or high triglycerides [HTG], respectively); and (b) were or were not on statin therapy (see S1 Fig). A triglyceride concentration of 1.70 mmol/L represents the upper level of normal range according to the National Cholesterol Education Program’s Adult Treatment Panel III recommendations [32].

Data are expressed as median (interquartile range) and/or mean±SD, for continuous variables, and number of cases and percentage for categorical variables. Continuous variables were compared by one-way ANOVA, for normally distributed variables, or Kruskall-Wallis test, in case of variables with a skewed distribution. Pearson ^2^ was applied to categorical variables. For post-hoc multiple comparisons, Scheffe’s test, Mann-Whitney U test, and ^2^ test, 1df were used for parametric, non-parametric, and categorical variables, respectively.

Binary logistic regression analyses with backward conditional entering of independent variables (probability for removal >0.10) were then applied. The outcome measures for microvascular complications were albuminuria and eGFR categories or CKD phenotypes for diabetic nephropathy, and any and advanced retinopathy for DR. In Model 1, the independent variable was a combination of two dichotomous variables (2x2 factorial combination), i.e. triglyceride levels (< or ±1.7 mmol/L) and statin treatment (presence or absence). In Models 2 and 3, the association of triglycerides below or above the threshold with microvascular complications was analysed separately in subjects not treated or treated with statins, respectively. The above analyses were repeated with substitution of deciles of triglyceride levels for triglycerides < or ±1.7 mmol/L. Covariates for all three models were age, male gender, smoking status, diabetes duration, HbA_1c_, BMI, HDL cholesterol, hypertension, and blockade of the renin-angiotensin system, and, in separate analyses, total or LDL cholesterol. Results of these analyses were expressed as odd ratios (ORs) with their 95% confidence intervals.

To test how the models fit the data, the Hosmer-Lemeshow test for logistic regression was applied. Finally, the quadratic regression analysis was employed to assess non-linearity between TG deciles (expressed as the median value of TG concentration within each decile) and the corresponding ORs for each microvascular endpoint. The F statistics was run to test whether addition of the quadratic term improved significantly the model’s ability to explain the variance of the dependent variable.

All p values were two-sided, and a p value of less than 0.05 was considered statistically significant. Statistical analyses were performed using SPSS version 13.0 (SPSS Inc., Chicago, Illinois, USA).

## Results

Of the 15,773 RIACE participants, 67.8% had triglyceride levels <1.7 mmol/L, whereas the remaining 32.2% had values ±1.7 mmol/L; in addition, 42.4% were on statin treatment, 27.7% among NTG and 14.7% among HTG subjects (see S1 Fig, S1 Table and S2 Table). Only 396 subjects (2.5%) were on fibrate treatment, 139 NTG and 257 HTG (1.3 vs. 5.1%, *P*<0.0001).

### Patients’ characteristics by triglyceride level and statin treatment

HTG subjects, either with or without statin, had higher HbA_1c_, BMI, waist circumference, total, LDL and non-HDL cholesterol, and albuminuria, and lower HDL cholesterol and, only for subjects on statin, eGFR, as compared with NTG individuals. Patients on statins were older, less frequently males and current smokers, and more frequently hypertensive and on anti-hypertensive treatment and therapy with blockers of the renin-angiotensin system, as compared with the corresponding individuals not taking statins (see S1 Table). Moreover, HTG patients, particularly if on statins, had higher prevalence of CKD, especially the albuminuric forms, than NTG subjects. In contrast, CVD and advanced DR were more prevalent in subjects on statin than in those not, independently of triglyceride levels (see S2 Table).

### Hypertriglyceridemia, with or without statin treatment, as an independent correlate of microvascular outcomes

Logistic regression analyses (Tables 1–3) showed that HTG, with or without statin, was independently associated with micro and macroalbuminuria, mildly to severely reduced eGFR, and all CKD phenotypes, especially Stages 3–5 albuminuric (Model 1). The increase in OR in HTG vs. NTG was lower in subjects not on statins (Model 2) than in those on statins (Model 3). In contrast, HTG was not positively associated with any or advanced DR (Table 4). Several covariates entered the models (see Tables 1–4 for Model 1).

**Table 1:** Independent association of triglyceride levels and statin treatment with microalbuminuria and macroalbuminuria in the RIACE study.*

	Microalbuminuria (n = 3,497)			Macroalbuminuria (n = 738)		
	OR	95% CI	*P*	OR	95% CI	*P*
**Independent variable**						
**Model 1 (TG—statin combination)**			<0.0001			<0.0001
**TG <1.70 mmol/L—no statin**	1.0			1.0		
**TG <1.70 mmol/L—statin**	0.869	0.785–0.960	0.006	0.984	0.790–1.225	0.884
**TG ≥1.70 mmol/L—no statin**	1.228	1.093–1.378	0.001	1.877	1.492–2.362	<0.0001
**TG ≥1.70 mmol/L—statin**	1.191	1.054–1.345	0.005	2.619	2.108–3.255	<0.0001
**Model 2 (TGs in not statin users)**						
**TG <1.70 mmol/L**	1.0			1.0		
**TG** **±** **1.70 mmol/L**	1.210	1.074–1.366	0.002	1.715	1.342–2.192	<0.0001
**Model 3 (TGs in statin users)**						
**TG <1.70 mmol/L**	1.0			1.0		
**TG** **±** **1.70 mmol/L**	1.428	1.255–1.624	<0.0001	2.592	2.060–3.260	<0.0001
**Covariates (Model 1)**						
**Age, x year**	1.022	1.018–1.027	<0.0001	1.023	1.014–1.033	<0.0001
**Male gender**	2.008	1.834–2.199	<0.0001	2.594	2.159–3.117	<0.0001
**Smoking**			<0.0001			<0.0001
**Never**	1.0			1.0		
**Former**	1.111	1.012–1.220	0.028	1.350	1.122–1.624	0.002
**Current**	1.353	1.206–1.518	<0.0001	1.778	1.422–2.223	<0.0001
**Diabetes duration, x year**	1.007	1.002–1.011	0.003	1.019	1.010–1.027	<0.0001
**HbA**_**1c**_ **, x 1% increment**	1.163	1.133–1.195	<0.0001	1.159	1.102–1.219	<0.0001
**BMI, x 1 unit**	1.023	1.014–1.031	<0.0001	1.042	1.025–1.058	<0.0001
**HDL cholesterol, x 0.03 mmol/L**	0.994	0.991–0.997	<0.0001	—-	—-	—-
**Hypertension**	1.314	1.137–1.517	<0.0001	2.419	1.611–3.630	<0.0001
**RAS blockade**	1.584	1.437–1.745	<0.0001	1.964	1.601–2.410	<0.0001
**DR**			<0.0001			<0.0001
**No**	1.0			1.0		
**Non-advanced**	1.410	1.254–1.584	<0.0001	1.882	1.512–2.343	<0.0001
**Advanced**	2.132	1.879–2.420	<0.0001	4.679	3.821–5.729	<0.0001

* Binary logistic regression analysis with backward conditional entering of independent variables.

RIACE = Renal Insufficiency And Cardiovascular Events; OR = odd ratio; CI = confidence interval; TG = triglyceride; RAS = renin-angiotensin system; DR = diabetic retinopathy.

**Table 2:** Role of triglyceride levels & statin treatment as covariates of categories of reduced eGFR in the RIACE study. *

	eGFR 60–89 ml/min/1.73 m^2^ (n = 8,152)				eGFR 30–59 ml/min/1.73 m^2^ (n = 2,701)			eGFR <30 ml/min/1.73 m^2^ (n = 258)			
	OR	95% CI	*p*		OR	95% CI	*p*	OR	95% CI		*p*
**Independent variable**											
**Model 1 (TG—statin combination)**					<0.0001					<0.0001	
**TG <1.70 mmol/L—no statin**	1.0				1.0			1.0			
**TG <1.70 mmol/L—statin**	1.147	1.045–1.259	0.004		1.363	1.178–1.578	<0.0001	1.327	0.883–1.994		0.173
**TG ≥1.70 mmol/L—no statin**	1.176	1.050–1.316	0.005		1.820	1.527–2.169	<0.0001	3.335	2.183–5,096		<0.0001
**TG ≥1.70 mmol/L—statin**	1.442	1.271–1.636	<0.0001		2.639	2.210–3.151	<0.0001	3.952	2.582–6.050		<0.0001
**Model 2 (TGs in not statin users)**											
**TG <1.70 mmol/L**	1.0				1.0			1.0			
**TG** **±** **1.70 mmol/L**	1.175	1.047–1.318	0.006		1.779	1.486–2.131	<0.0001	3.515	2.263–5.461		<0.0001
**Model 3 (TGs in statin users)**											
**TG <1.70 mmol/L**	1.0				1.0			1.0			
**TG** **±** **1.70 mmol/L**	1.265	1.102–1.451	0.001		1.992	1.646–2.411	<0.0001	2.770	1.743–4.405		<0.0001
**Covariates (Model 1)**											
**Age, x year**	1.061	1.056–1.065	<0.0001	1.131		1.122–1.140	<0.0001	1.153	1.131–1.176		<0.0001
**Male gender**	0.782	0.719–0.851	<0.0001	0.505		0.443–0.574	<0.0001	0.526	0.381–0.726		<0.0001
**Smoking**			<0.0001				0.009				0.038
**Never**	1.0			1.0				1.0			
**Former**	1.078	0.983–1.183	0.108	1.109		0.965–1.276	0.146	1.307	0.918–1.861		0.137
**Current**	0.832	0.748–0.926	0.001	0.820		0.683–0.984	0.033	1.725	1.118–2.663		0.014
**Diabetes duration, x year**	1.007	1.002–1.011	0.004	1.014		1.008–1.021	<0.0001	1.041	1.026–1.057		<0.0001
**HbA**_**1c**_ **, x 1% increment**	0.948	0.923–0.974	<0.0001	—-		—-	—-	0.902	0.816–0.997		0.044
**BMI, x 1 unit**	—-	—-	—-	1.022		1.010–1.034	<0.0001	1.058	1.030–1.088		<0.0001
**HDL cholesterol, x 0.03 mmol/L**	0.994	0.991–0.997	<0.0001	0.981		0.977–0.986	<0.0001	0.977	0.965–0.990		<0.0001
**Hypertension**	—-	—-	—-	1.537		1.235–1.913	<0.0001	2.362	1.160–4.808		0.018
**RAS blockade**	1.186	1.097–1.282	<0.0001	1.669		1.451–1.920	<0.0001	1.585	1.109–2.264		0.011
**DR**			0.088				<0.0001				<0.0001
**No**	1.0			1.0				1.0			
**Non-advanced**	0.887	0.785–1.003	0.057	1.103		0.928–1.311	0.267	1.719	1-150-2.568		0.008
**Advanced**	1.058	0.917–1.220	0.442	1.776		1.468–2.150	<0.0001	4.967	3.427–7.200		<0.0001

* Binary logistic regression analysis with backward conditional entering of independent variables.

RIACE = Renal Insufficiency And Cardiovascular Events; eGFR = estimated glomerular filtration rate; OR = odd ratio; CI = confidence interval; TG = triglyceride; RAS = renin-angiotensin system; DR = diabetic retinopathy.

**Table 3:** Role of triglyceride levels & statin treatment as covariates of CKD phenotypes in the RIACE study. *

	CKD Stages 1–2 (n = 2,949)			CKD Stages ≥3 non albuminuric (n = 1,673)			CKD Stages ≥3 albuminuric (n = 1,286)		
	OR	95% CI	p	OR	95% CI	p	OR	95% CI	p
**Independent variable**									
**Model 1 (TG—statin combination)**			<0.0001			<0.0001			<0.0001
**TG <1.70 mmol/L—no statin**	1.0			1.0			1.0		
**TG <1.70 mmol/L—statin**	0.875	0.784–0.976	0.016	1.182	1.027–1.361	0.020	1.033	0.870–1.226	0.710
**TG ≥1.70 mmol/L—no statin**	1.227	1.081–1.393	0.002	1.535	1.294–1.820	<0.0001	2.003	1.654–2.427	<0.0001
**TG ≥1.70 mmol/L—statin**	1.246	1.090–1.426	0.001	1.838	1.553–2.175	<0.0001	2.667	2.213–3.214	<0.0001
**Model 2**									
**TG <1.70 mmol/L**	1.0			1.0			1.0		
**TG** **±** **1.70 mmol/L**	1.205	1.058–1.373	0.005	1.499	1.259–1.786	<0.0001	1.960	1.606–2.392	<0.0001
**Model 3**									
**TG <1.70 mmol/L**	1.0			1.0			1.0		
**TG** **±** **1.70 mmol/L**	1.452	1.259–1.675	<0.0001	1.559	1.304–1.864	<0.0001	2.631	2.151–3.219	<0.0001
**Covariates (Model 1)**									
**Age, x year**	1.019	1.014–1.024	<0.0001	1.101	1.093–1.109	<0.0001	1.095	1.086–1.105	<0.0001
**Male gender**	2.067	1.868–2.288	<0.0001	0.469	0.416–0.530	<0.0001	1.373	1.186–1.589	<0.0001
**Smoking**			<0.0001	—-	—-	—-			0.001
**Never**	1.0						1.0		
**Former**	1.107	0.999–1.227	0.052				1.300	1.118–1.510	0.001
**Current**	1.429	1.266–1.613	<0.0001				1.263	1.031–1.548	0.024
**Diabetes duration, x year**	1.006	1.001–1.011	0.016	1.007	1.002–1.013	0.011	1.020	1.013–1.027	<0.0001
**HbA**_**1c**_ **, x 1% increment**	1.197	1.162–1.232	<0.0001	—-	—-	—-	1.108	1.061–1.157	<0.0001
**BMI, x 1 unit**	1.031	1.022–1.040	<0.0001	1.026	1.015–1.038	<0.0001	1.030	1.016–1.044	<0.0001
**HDL cholesterol, x 0.03 mmol/L**	0.995	0.992–0.999	0.009	0.984	0.979–0.988	<0.0001	0.982	0.977–0.988	<0.0001
**Hypertension**	1.367	1.173–1.592	<0.0001	1.367	1.096–1.706	0.006	1.634	1.213–2.201	0.001
**RAS blockade**	1.652	1.487–1.835	<0.0001	1.466	1.283–1.675	<0.0001	2.128	1.807–2.506	<0.0001
**DR**			<0.0001			<0.0001			<0.0001
**No**	1.0			1.0			1.0		
**Non-advanced**	1.348	1.184–1.535	<0.0001	1.096	0.919–1.306	0.308	1.925	1.613–2.297	<0.0001
**Advanced**	2.256	1.960–2.596	<0.0001	1.462	1.200–1.780	<0.0001	4.110	3.429–4.927	<0.0001

* Binary logistic regression analysis with backward conditional entering of independent variables.

RIACE = Renal Insufficiency And Cardiovascular Events; CKD = chronic kidney disease; OR = odd ratio; CI = confidence interval; TG = triglyceride; RAS = renin-angiotensin system; DR = diabetic retinopathy.

**Table 4:** Independent association of triglyceride levels and statin treatment with any and advanced DR in the RIACE study. *

	Any DR (n = 3,497)			Advanced DR (n = 1,540)		
	OR	95% CI	*P*	OR	95% CI	*P*
**Independent variable**						
**Model 1 (TG—statin combination)**			0.001			0.015
**TG <1.70 mmol/L—no statin**	1.0			1.0		
**TG <1.70 mmol/L—statin**	1.049	0.949–1.159	0.348	1.081	0.939–1.244	0.279
**TG ≥1.70 mmol/L—no statin**	0.805	0.712–0.910	0.001	0.807	0.679–0.958	0.014
**TG ≥1.70 mmol/L—statin**	0.932	0.822–1.056	0.269	1.004	0.846–1.191	0.966
**Model 2 (TGs in not statin users)**						
**TG ≥1.70 mmol/L**	1.0			1.0		
**TG** **±** **1.70 mmol/L**	0.835	0.738–0.944	0.004	0.857	0.721–1.018	0.080
**Model 3 (TGs in statin users)**						
**TG ≥1.70 mmol/L**	1.0			1.0		
**TG** **±** **1.70 mmol/L**	0.853	0.745–0.976	0.021	0.894	0.744–1.073	0.227
**Covariates (Model 1)**						
**Age, x year**	0.983	0.978–0.988	<0.0001	0.970	0.963–0.976	<0.0001
**Male gender**	1.079	0.987–1.180	0.093	—-	—-	—-
**Smoking**			0.094			0.001
**Never**	1.0			1.0		
**Former**	0.910	0.826–1.004	0.059	0.839	0.736–0.957	0.009
**Current**	0.906	0.802–1.024	0113	0.761	0.639–0.905	0.002
**Diabetes duration, x year**	1.067	1.063–1.072	<0.0001	1.067	1.060–1.073	<0.0001
**HbA**_**1c**_ **, x 1% increment**	1.206	1.175–1.239	<0.0001	1.179	1.138–1.221	<0.0001
**BMI, x 1 unit**	1.010	1.002–1.019	0.014	1.011	1.000–1.022	0.051
**HDL cholesterol, x 0.03mmol/L**	—-	—-	—-	—-	—-	—-
**Hypertension**	—-	—-	—-	1.200	0.968–1.487	0.096
**RAS blockade**	1.479	1.354–1.616	<0.0001	1.425	1.237–1.643	<0.0001
**Albuminuria category**			<0.0001			<0.0001
**Normoalbuminuria**	1.0			1.0		
**Microalbuminuria**	1.646	1.498–1.809	<0.0001	2.046	1.803–2.323	<0.0001
**Macroalbuminuiria**	2.609	2.195–3.101	<0.0001	3.876	3.152–4.764	<0.0001
**eGFR category**			<0.0001			<0.0001
**≥90 ml/min/1.73 m** ^**2**^	1.0			1.0		
**60–89 ml/min/1.73 m** ^**2**^	0.977	0.884–1.080	0.650	1.095	0.948–1.265	0.219
**30–59 ml/min/1.73 m** ^**2**^	1.229	1.078–1.400	0.002	1.529	1.278–1.830	<0.0001
**<30 ml/min/1.73 m** ^**2**^	1.697	1.262–2.280	<0.0001	2.414	1.694–3.439	<0.0001

* Binary logistic regression analysis with backward conditional entering of independent variables.

DR = diabetic retinopathy; RIACE = Renal Insufficiency And Cardiovascular Events; OR = odd ratio; CI = confidence interval; TG = triglyceride; RAS = renin-angiotensin system; eGFR = estimated glomerular filtration rate.

When analyses were repeated adding total or LDL cholesterol as covariates, these variables entered the model only for macroalbuminuria and eGFR <30 ml/min/1.73 m^2^ (total cholesterol) or 60–89 ml/min/1.73 m^2^ (LDL cholesterol) and the independent association of triglyceride levels and statin treatment with microvascular complications was not influenced (data not shown). Also exclusion of subjects on fibrate therapy did not affects results (data not shown). The Hosmer-Lemeshow test was significant only for eGFR 60–89 ml/min/1.73 m^2^ in Model 1 (χ^2^ = 17.638; 8 df, p = 0.024) and 2 (χ^2^ = 25.656; 8 df, p = 0.001), and for any DR, again in Model 1 (χ^2^ = 40.501; 8 df, p<0.001) and 2 (χ^2^ = 55.057, 8 df, p<0.001).

### Relation of triglyceride levels with microvascular disease

**Fig 1:**
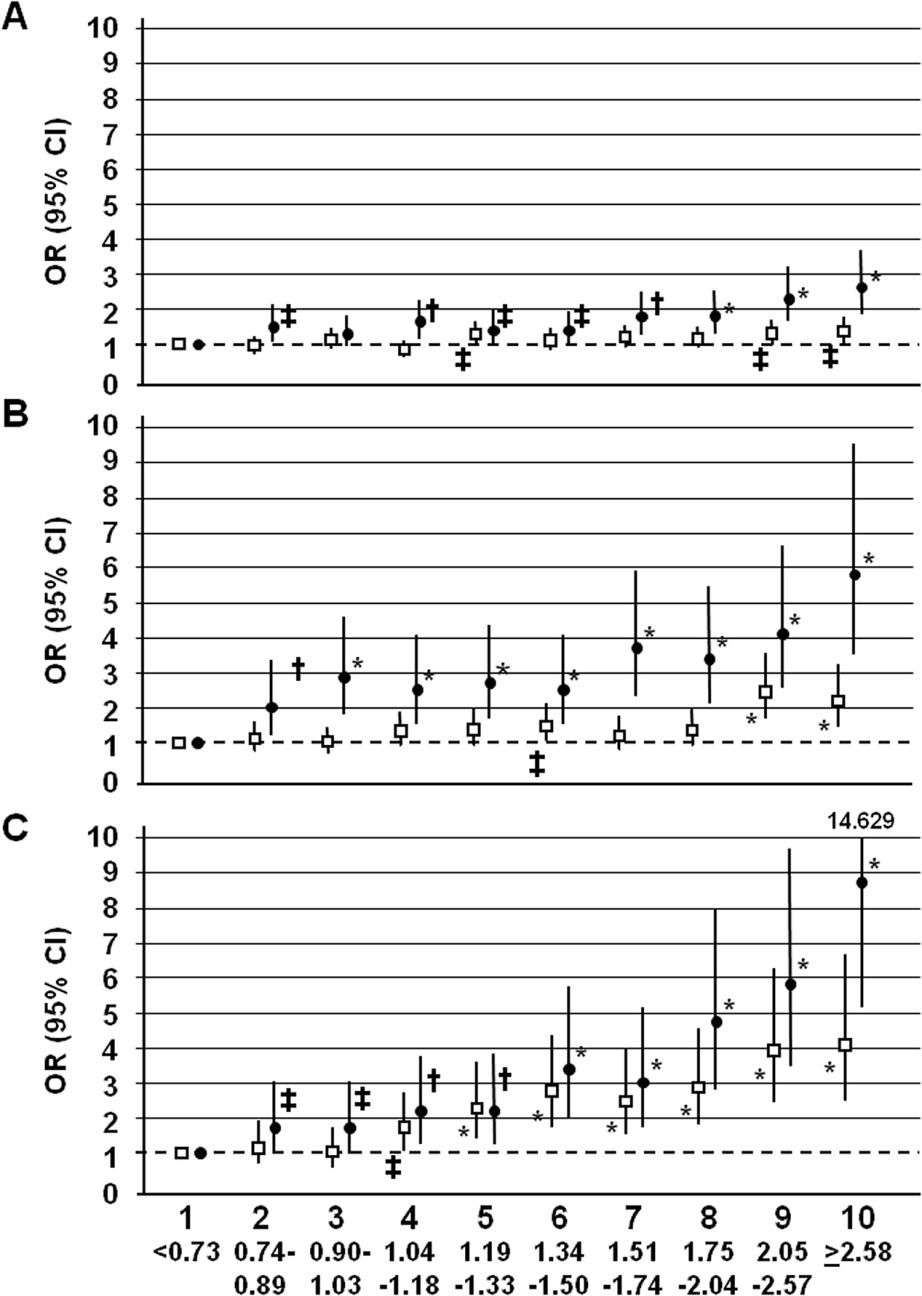
Adjusted risk of CKD Stages 1–2 (Panel A), CKD Stages 3–5 nonalbuminuric (Panel B) and CKD Stages 3–5 albuminuric (Panel C) (OR [95% CI]) according to deciles of triglyceride levels in subjects without (□) and with (●) statin treatment. ORs are adjusted for age, male gender, smoking status, diabetes duration, HbA_1c_, BMI, HDL and LDL cholesterol, hypertension, and blockade of the renin-angiotensin system. Significant at * *P*<0.0001, † *P* at least <0.01, ‡ *P* at least <0.05.

The OR for all measures of CKD (Table 5), but not for any or advanced DR (not shown), adjusted for several variables including LDL cholesterol, increased for every 0.26 mmol/L increase (approximately one decile) in triglyceride levels. The increase in OR was higher with increasing severity of albuminuria, eGFR loss and CKD phenotype as well as in subjects on statin treatment than in those not treated with these agents. Risk for CKD increased with deciles of triglyceride levels. In subjects not on statin therapy, excess risk was significant for triglyceride values > 2.05 mmol/L, for CKD Stages 1–2 and CKD Stages ≥3 nonalbuminuric, and >1.05 mmol, for CKD Stages ≥3 albuminuric. In contrast, in subjects on statin therapy, the threshold was very low (>0,74 mmol/L) for all CKD phenotypes (Fig 1). Increases were linear, since adding the quadratic term to the regression equation did not change significantly the results (not shown).

**Table 5:** Odds ratios corresponding to a difference of one decile in triglycerides levels for measures of CKD in all subjects and in those without and with statin treatment. *

Variable	OR	95% CI	*P*
**All subjects**			
**Microalbuminuria**	1.062	1.046–1.079	<0.0001
**Macroalbuminuria**	1.181	1.146–1.217	<0.0001
**eGFR 60–89 ml/min/1.73 m** ^**2**^	1.047	1.032–1.063	<0.0001
**eGFR 30–59 ml/min/1.73 m** ^**2**^	1.151	1.125–1.178	<0.0001
**eGFR <30 ml/min/1.73 m** ^**2**^	1.354	1.272–1.442	<0.0001
**CKD Stages 1–2**	1.068	1.051–1.085	<0.0001
**CKD Stages** **±** **3 nonalbuminuric**	1.110	1.086–1.136	<0.0001
**CKD Stages** **±** **3 albuminuric**	1.204	1.172–1.236	<0.0001
**Subjects not on statin therapy**			
**Microalbuminuria**	1.050	1.028–1.071	<0.0001
**Macroalbuminuria**	1.169	1.122–1.218	<0.0001
**eGFR 60–89 ml/min/1.73 m** ^**2**^	1.040	1.021–1.060	<0.0001
**eGFR 30–59 ml/min/1.73 m** ^**2**^	1.142	1.107–1.179	<0.0001
**eGFR <30 ml/min/1.73 m** ^**2**^	1.408	1.303–1.522	<0.0001
**CKD Stages 1–2**	1.046	1.023–1.069	<0.0001
**CKD Stages** **±** **3 nonalbuminuric**	1.088	1.055–1.122	<0.0001
**CKD Stages** **±** **3 albuminuric**	1.184	1.141–1.228	<0.0001
**Subjects on statin therapy**			
**Microalbuminuria**	1.081	1.057–1.106	<0.0001
**Macroalbuminuria**	1.192	1.142–1.245	<0.0001
**eGFR 60–89 ml/min/1.73 m** ^**2**^	1.064	1.041–1.088	<0.0001
**eGFR 30–59 ml/min/1.73 m** ^**2**^	1.173	1.133–1.214	<0.0001
**eGFR <30 ml/min/1.73 m** ^**2**^	1.315	1.197–1.444	<0.0001
**CKD Stages 1–2**	1.087	1.060–1.115	<0.0001
**CKD Stages** **±** **3 nonalbuminuric**	1.131	1.094–1.169	<0.0001
**CKD Stages** **±** **3 albuminuric**	1.242	1.198–1.288	<0.0001

* Binary logistic regression analysis with backward conditional entering of independent variables. Other covariates were age, male gender, smoking status, diabetes duration, HbA_1c_, BMI, HDL and LDL cholesterol, hypertension, and blockade of the renin-angiotensin system.

## Discussion

This study reports an association of hypertriglyceridemia with CKD, but not DR, in subjects with type 2 diabetes. In these individuals, risk of CKD increased by 7–20% for every decile increase in triglyceride concentrations. The relationship between triglycerides and CKD was stronger with increasing severity of albuminuria, eGFR loss and CKD phenotype and was independent of statin treatment. In fact, the burden of CKD was higher in HTG than in NTG individuals, with and without statins, though subjects on statins carried a higher risk than those not treated with these agents (9–24% vs. 5–18% increase per decile). Excess risk was significant for triglyceride values within the normal range in statin-treated patients.

These results are consistent with the literature, showing more frequently an association of triglyceride levels with CKD than with DR [15–18]. They also confirm and extend the findings of a recent multicenter cross-sectional case-control study, in which however CKD and DR were variably defined and not graded [33]. Interestingly, this study reported that the OR for CKD increased by 23% for every 0.5mmol/L (approximately 1 quintile) increase in triglycerides, a figure quite similar to ours, whereas DR was significantly associated with triglycerides in matched analysis but not after additional adjustment.

Our data are also in keeping with studies in experimental animals fed a high fat diet which suggested that lipotoxicity might represent a major mechanism in renal disease by inducing glomerulosclerosis and tubulo-interstitial injury, associated with renal lipid accumulation [34]. Deposition of triglycerides in renal parenchimal cells results from both increased glomerular uptake of circulating lipids or tubular reabsorption of albumin-bound fatty acids [34] and enhanced renal fatty acid synthesis via a sterol regulatory element binding protein 1c-dependent pathway [35]. Cellular lipids undergo chemical modification, particularly oxidation, with formation of advanced lipoxidation endproducts, which exert injurious effect both directly and via receptor-mediated pathways [36].

However, dyslipidaemia characterized by increased triglyceride levels, and to a lesser extent reduced HDL cholesterol, is a known consequence of renal dysfunction, especially when eGFR falls below 60 ml/min/1.73m^2^ [37], and is associated with adverse renal outcomes in CKD Stages 3–5 [38]. This suggests that, in a cross-sectional analysis, the association between triglycerides and CKD might reflect the bidirectional nature of this relation. The finding of a significant association between triglycerides and CKD, but not DR, seems to support this view. However, this observation might also reflect the fact that lipid extravasation is restricted in the retina by the blood-retinal barrier and that disruption of this barrier, rather than increased lipid levels, is required for lipid-induced retinal injury [39]. Moreover, a significant relation between triglycerides and CKD was detected also for degrees of renal dysfunction which are not accompanied by significant lipid abnormalities, such as CKD Stages 1–2 and eGFR 60–89 ml/min/1.73m^2^. Yet, this relation was stronger with increasing severity of CKD, as defined by albuminuria and eGFR category or CKD phenotype, thus suggesting that it reflects also the impact of impaired renal function on lipid profile.

Taken together, these data emphasize the role of triglycerides in the development of renal disease associated with type 2 diabetes, though this relationship is bi-directional. In addition, they suggest that statin therapy might be useful in diabetic subjects also by virtue of its ability to reduce triglyceride levels. Results from our study and from that of Sacks *et al* [33] also suggest that data from the Action to Control Cardiovascular Risk in Diabetes Lipid Trial and the Fenofibrate Intervention and Event Lowering in Diabetes Study [19–21] should be revisited. In fact, targeting triglycerides might be effective in preventing or delaying loss of renal function in diabetic individuals beyond the stimulation of peroxisome proliferator-activated receptor-α induced by fibrates, which might reduce renal lipid synthesis [34]. This view is supported by the independent association between elevated triglyceride levels and measures of CKD, though data from statin-treated patients do not allow to draw conclusive remarks. In fact, this association was independent from statin treatment. Moreover, in a cross-sectional analysis, the higher CKD risk in subjects with than in those without statin therapy within each triglyceride category (NTG or HTG) or decile may raise different interpretations. On the one hand, it may indicate that statin treatment is not very effective in reducing CKD risk in diabetic patients, as also suggested by the observation that excess risk was significant for triglyceride values within the normal range in statin-treated subjects. Furthermore, it might reflect reverse causality, since CKD is associated with dyslipidemia which requires statin treatment. On the other hand, the fact that ORs for measures of CKD were lower in NTG subjects on statins than in HTG individuals not on statins, might indicate that statin treatment is effective, since NTG patients on statins likely had high triglycerides before starting statin therapy, in keeping with the observation that the higher are triglyceride levels at baseline the higher is the extent of their reduction with statins [40].

Strengths of this study include the large size of the cohort, the completeness of data, the analysis of a contemporary dataset, and particularly the standardized definition and grading of microvascular complications and the dissection of the effect of statin treatment. The latter was facilitated by the characteristics of the cohort, in which less than half of subjects were on statins (and only a few on fibrates), as reported in other cohorts [23,41]. The main limitation is the cross-sectional design of the study which did not allow to exclude reverse causality that might have affected the analysis of the bi-directional relationship between triglyceride levels or statin treatment and CKD. Potential limitations concerning assessment of CKD and DR, including non-centralized measurements of albuminuria and serum creatinine and the use of funduscopy have been extensively addressed in previous RIACE reports [25,26,30,31].

In conclusion, this cross-sectional analysis of the large RIACE cohort showed that hypertriglyceridemia is associated with renal, but not retinal, complications in subjects with type 2 diabetes. This relationship was independent of statin treatment, which is known to decrease triglyceride levels, and excess risk was observed for triglyceride values within the normal range in statin-treated patients.

## Supporting Information

S1 Fig Percent of patients with triglyceride levels <1.7 mmol/L and ±1.7 mmol/L, without (open bars) and with (closed bars) statin treatment. Values on top of columns are n (%).
(DOC)

S1 RIACE Investigators Participating diabetes centers. (DOC)

S1 Table Clinical characteristics of type 2 diabetic subjects from the RIACE cohort stratified by triglyceride levels and statin treatment. (DOC)

S2 Table Microvascular complications and cardiovascular disease in type 2 diabetic subjects from the RIACE cohort stratified by triglyceride levels and statin treatment. (DOC)
