# Supplementary material for: Hypertriglyceridemia Is Independently Associated with Renal, but Not Retinal Complications in Subjects with Type 2 Diabetes: A Cross-Sectional Analysis of the Renal Insufficiency And Cardiovascular Events (RIACE) Italian Multicenter Study
Source: PLoS One. 2015 May 5;10(5):e0125512. doi: 10.1371/journal.pone.0125512 (PMC4420503; doi:10.1371/journal.pone.0125512)

**S1_Fig.doc.** Percent of patients with triglyceride levels <1.7 mmol/L and >1.7 mmol/L, without (open bars) and with (closed bars) statin treatment. Values on top of columns are n (%).


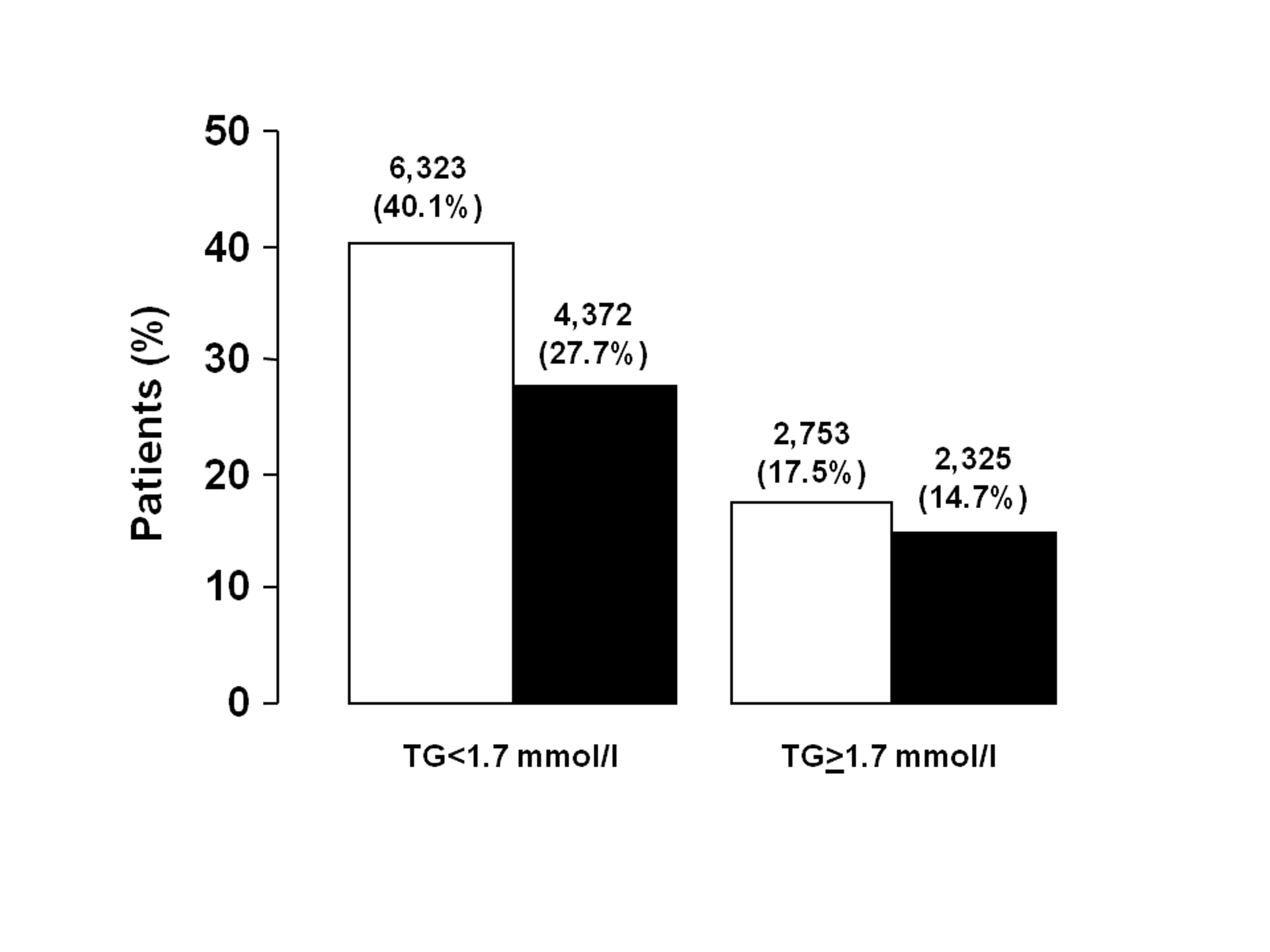

Supplement: S1 Fig — Values on top of columns are n (%). (DOC) [file pone.0125512.s001.doc]
