# Supplementary material for: Hypertriglyceridemia Is Independently Associated with Renal, but Not Retinal Complications in Subjects with Type 2 Diabetes: A Cross-Sectional Analysis of the Renal Insufficiency And Cardiovascular Events (RIACE) Italian Multicenter Study
Source: PLoS One. 2015 May 5;10(5):e0125512. doi: 10.1371/journal.pone.0125512 (PMC4420503; doi:10.1371/journal.pone.0125512)
Supplement: S1 RIACE Investigators — (DOC) [file pone.0125512.s002.doc]

**S1_RIACE Investigators.** Participating diabetes centres.

1. Azienda Ospedaliera Sant'Andrea, Roma (Coordinating Center): Giuseppe Pugliese, Laura Salvi, Lucilla Bollanti, Alessandra Bazuro and Carla Maccora.
2. Ospedale Le Molinette, Torino: Paolo Cavallo-Perin, Gabriella Gruden and Bartolomeo Lorenzati.
3. Ospedale San Luigi Gonzaga, Orbassano: Mariella Trovati, Franco Cavalot, Manuela Valle, Leonardo di Martino and Fabio Mazzaglia.
4. Ospedale San Raffaele, Milan: Giampaolo Zerbini, Valentina Martina, Silvia Maestroni and Valentina Capuano.
5. IRCCS “Cà Granda – Ospedale Maggiore Policlinico”, Milan: Emanuela Orsi, Laura Montefusco and Dario Zimbalatti.
6. Ospedale San Paolo, Milan: Antonio Pontiroli, Annamaria Veronelli and Barbara Zecchini.
7. Ospedale San Giuseppe, Milan: Maura Arosio and Alessia Dolci.
8. Ospedali Riuniti, Bergamo: Roberto Trevisan and Anna Corsi.
9. Ospedale Maggiore, Verona: Enzo Bonora and Giacomo Zoppini.
10. Policlinico Universitario, Padova: Angelo Avogaro, Monica Vedovato and Elisa Pagnin.
11. Ospedale Cisanello, Azienda Ospedaliero-Universitaria Pisana, Pisa: Giuseppe Penno, Laura Pucci, Daniela Lucchesi, Eleonora Russo and Monia Garofolo.
12. Ospedale Santa Chiara, Azienda Ospedaliero-Universitaria Pisana, Pisa: Anna Solini.
13. Ospedale Le Scotte, Siena: Francesco Dotta, Cecilia Fondelli and Laura Nigi.
14. Policlinico Umberto I, Roma: Susanna Morano and Alessandra Gatti.
15. Ospedale S. Maria Goretti, Latina: Raffaella Buzzetti.
16. Ospedali Riuniti, Foggia: Mauro Cignarelli, Olga Lamacchia, Sabina Pinnelli and Lucia Monaco.
17. Policlinico Universitario, Bari: Francesco Giorgino, Luigi Laviola and Sebastio Perrini.
18. Policlinico Mater Domini, Catanzaro: Giorgio Sesti and Francesco Andreozzi.
19. Policlinico Monserrato, Cagliari: Marco Giorgio Baroni and Giuseppina Frau.
