# Supplementary material for: Hypertriglyceridemia Is Independently Associated with Renal, but Not Retinal Complications in Subjects with Type 2 Diabetes: A Cross-Sectional Analysis of the Renal Insufficiency And Cardiovascular Events (RIACE) Italian Multicenter Study
Source: PLoS One. 2015 May 5;10(5):e0125512. doi: 10.1371/journal.pone.0125512 (PMC4420503; doi:10.1371/journal.pone.0125512)
Supplement: S1 Table — (DOC) [file pone.0125512.s003.doc]

**S1_Table.** Clinical characteristics of type 2 diabetic subjects from the RIACE cohort stratified by triglyceride levels and statin treatment.

| **Variables** | **Triglycerides levels <1.70 mmol/L** | | Triglycerides level ≥1.70 mmol/L | | *P* |
| --- | --- | --- | --- | --- | --- |
|  | **no statin treatment** | **statin treatment** | **no statin treatment** | **statin treatment** |  |
| **n (%)** | 6,323 (40.1) | 4,372 (27.7) | 2,753 (17.5) | 2,325 (14.7) |  |
| **Age, years** | 66.0±11.1 | 67.5±8.9 * | 63.8±11.3 * | 66.0±9.0 | <0.0001 |
| **Male gender, n (%)** | 3,631 (57.4) | 2,475 (56.6) | 1,594 (57.9) | 1,260 (54.2) ‡ | 0.031 |
| **Smoking, n (%)** |  |  |  |  | <0.0001 |
| **Never** | 3,757 (59.4) † | 2,470 (56.5) † | 1,486 (54.0) | 1,215 (52.3) |  |
| **Former** | 1,641 (26.0) | 1,296 (29.6) || | 771 (28.0) | 726 (31.2) || |  |
| **Current** | 925 (14.6) | 606 (13.9) | 496 (18.0) § | 384 (16.5) || |  |
| **Age at diabetes diagnosis, years** | 53.0±11.7 | 52.9±11.1 | 52.1±11.3 ‡ | 53.3±11.1 | 0.001 |
| Diabetes duration, years | 13.0±10.5 | 14.6±10.2 * | 11.6±9.6 * | 12.8±9.4 | <0.0001 |
| **HbA_1c_, % (mmol/mol)** | 7.39±1.43 | 7.42±1.31 | 7.84±1.74 § | 7.88±1.66 § | <0.0001 |
|  | (57.3±15.6) | (57.6±14.3) | (62.2±19.0) | (62.6±18.1) |  |
| **Anti-hyperglycemic treatment, n (%)** |  |  |  |  | <0.0001 |
| **Diet** | 997 (15.8) ‡ | 507 (11.6) | 369 (13.4) ‡ | 253 (10.9) |  |
| **OHA** | 3,801 (60.1) | 2,793 (63.9) || | 1,717 (62.4) || | 1,370 (58.9) |  |
| **OHA+insulin** | 515 (8.1) † | 424 (9.7) | 280 (10.2) | 300 (12.9) |  |
| **Insulin** | 1,010 (16.0) || | 648 (14.8) | 387 (14.1) | 402 (17.3) || |  |
| **BMI, kg/m^2^** |  |  |  |  |  |
| **Males** | 27.6±4.4 | 27.9±4.2 | 29.7±4.6 § | 29.7±4.3 § | <0.0001 |
| **Females** | 29.3±6.1 | 29.0±5.4 | 31.1±6.0 § | 30.7±5.4 § | <0.0001 |
| **Waist circumference, cm** |  |  |  |  |  |
| **Males** | 100.6±10.2 | 101.4±9.8 | 105.4±10.7 § | 105.4±10.2 § | <0.0001 |
| **Females** | 101.7±12.4 | 101.2±11.0 | 105.5±12.1 § | 104.8±11.3 § | <0.0001 |
| **Triglycerides, mmol/l** | 1.08 (0.83-1.33) | 1.13 (0.89-1.38) | 2.21 (1.92-2.78) | 2.23 (1.92-2.73) | NA |
| **Total cholesterol, mmol/l** | 4.75±0.88 * | 4.44±0.92 * | 5.25±1.03 * | 4.98±1.09 * | <0.0001 |
| **HDL cholesterol, mmol/l** |  |  |  |  |  |
| **Males** | 1.29±0.33 | 1.28±0.33 | 1.05±0.26 § | 1.09±0.26 § | <0.0001 |
| **Females** | 1.46±0.38 | 1.47±0.35 | 1.21±0.31 § | 1.39±0.37 § | <0.0001 |
| **LDL cholesterol, mmol/l** | 2.89±0.77 * | 2.56±0.80 * | 3.00±0.90 * | 2.70±0.96 * | <0.0001 |
| **Non-HDL cholesterol, mmol/l** | 3.39±0.81 * | 3.07±0.83 * | 4.13±0.97 * | 3.82±1.02 * | <0.0001 |
| **Dyslipidemia, n (%)** | 4,231 (66.9) | 4,372 (100.0) | 2,032 (73.8) | 2,325 (100.0) | <0.0001 ¶ |
| **Lipid-lowering treatment, n (%)** | 212 (3.4) | 4,372 (100.0) | 377 (13.7) | 2,325 (100.0) | <0.0001 ¶ |
| **Fibrates, n (%)** | 127 (2.0) | 12 (0.3) | 225 (8.2) * | 32 (1.4) | <0.0001 |
| **Systolic BP, mmHg** | 137.4±17.8 | 138.3±18.2 | 137.8±17.8 | 139.8±18.4 ‡ | <0.0001 |
| **Diastolic BP, mmHg** | 78.7±9.2 | 78.1±9.3 ‡ | 79.8±9.8 | 78.8±9.6 | <0.0001 |
| **Hypertension, n (%)** | 4,943 (78.2) † | 3,870 (88.5) † | 2,258 (82.0) † | 2,118 (91.1) † | <0.0001 |
| **Anti-hypertensive treatment, n (%)** | 3,925 (62.1) † | 3,464 (79.2) † | 1,841 (66.9) † | 1,920 (82.6) † | <0.0001 |
| **RAS blockers, n (%)** | 3,179 (50.3) * | 2,848 (65.1) * | 1,506 (54.7) * | 1,632 (70.2) * | <0.0001 |
| **Albuminuria, mg/24 hours** | 12.5 (6.2-28.2) | 12.5 (6.1-29.0) | 16.0 (7.6-43.1) § | 16.7 (7.8-53.8) § | <0.0001 |
| **Serum creatinine, μmol/l** | 79.6 (66.3-90.2) | 79.6 (68.1-92.8) | 79.6 (69.0-97.2) | 84.0 (70.7-102.5) § | <0.0001 |
| **eGFR, ml/min/1.73 m^2^** | 80.7 (67.4-95.3) | 77.8 (65.0-91.2) | 77.9 (62.9-93.8) | 72.3 (57.1-87.8) § | <0.0001 |

Values are mean±SD or median (interquartile range) for continuous variables and n (%) for categorical variables. *P* values for comparison among groups using the one-way ANOVA for parametric or the corresponding Kruskal-Wallis test for nonparametric (triglycerides, albuminuria, serum creatinine and eGFR) continuous variables, and the χ^2^ test, 3df for categorical variables. Post-hoc multiple comparison using the Scheffe’s test for parametric or the Mann-Whitney U test for nonparametric variables, and the χ^2^ test, 1df for categorical variables: * *P*<0.0001, † *P* at least <0.005, and ‡ *P* at least <0.05 vs. each other group; § *P* at least <0.0001, and || *P*<0.05 vs. groups with no symbols; ¶ comparison between no statin groups. RIACE = Renal Insufficiency And Cardiovascular Events; BP = blood pressure; RAS = renin-angiotensin system; eGFR = estimated glomerular filtration rate.
