# Supplementary material for: Hypertriglyceridemia Is Independently Associated with Renal, but Not Retinal Complications in Subjects with Type 2 Diabetes: A Cross-Sectional Analysis of the Renal Insufficiency And Cardiovascular Events (RIACE) Italian Multicenter Study
Source: PLoS One. 2015 May 5;10(5):e0125512. doi: 10.1371/journal.pone.0125512 (PMC4420503; doi:10.1371/journal.pone.0125512)
Supplement: S2 Table — (DOC) [file pone.0125512.s004.doc]

**S2_Table.** Microvascular complications and cardiovascular disease in type 2 diabetic subjects from the RIACE cohort stratified by triglyceride levels and statin treatment.

| **Variables** | **Triglycerides levels <1.70 mmol/l** | | Triglycerides level ≥1.70 mmol/l | | *P* |
| --- | --- | --- | --- | --- | --- |
|  | **no statin treatment** | **statin treatment** | **no statin treatment** | **statin treatment** |  |
| **n (%)** | 6,323 (40.1) | 4,372 (27.7) | 2,753 (17.5) | 2,325 (14.7) |  |
| **Albuminuria category** |  |  |  |  | <0.0001 |
| **Normoalbuminuria** | 4,826 (76.3) § | 3,309 (75.7) § | 1,898 (68.9) | 1,505 (64.7) |  |
| **Microalbuminuria** | 1,303 (20.6) | 893 (20.4) | 697 (25.3) § | 604 (26.0) § |  |
| **Macroalbuminuria** | 194 (3.1) | 170 (3.9) | 158 (5.7) § | 216 (9.3) * |  |
| **eGFR category** |  |  |  |  | <0.0001 |
| **≥90 ml/min/1.73 m^2^** | 2,117 (33.5) * | 1,181 (27.0) * | 853 (31.0) * | 511 (22.0) * |  |
| **60-89 ml/min/1.73 m^2^** | 3,282 (51.9) † | 2,394 (54.8) † | 1,315 (47.8) | 1,161 (49.9) |  |
| **30-59 ml/min/1.73 m^2^** | 856 (13.5) * | 743 (17.0) | 517 (18.8) | 585 (25.2) * |  |
| **<30 ml/min/1.73 m^2^** | 68 (1.1) | 54 (1.2) | 68 (2.5) § | 68 (2.9) § |  |
| **CKD phenotype** |  |  |  |  | <0.0001 |
| **no CKD** | 4,276 (67.6) * | 2,830 (64.5) * | 1,591 (57.8) * | 1,178 (50.7) * |  |
| **CKD Stages 1-2** | 1,123 (17.8) | 755 (17.3) | 577 (21.0) || | 494 (21.2) || |  |
| **CKD Stages >3 nonalbuminuric** | 550 (8.7) * | 489 (11.2) | 307 (11.2) | 327 (14.1) || |  |
| **CKD Stages >3 albuminuric** | 374 (5.9) | 308 (7.0) | 278 (10.1) § | 326 (14.0) § |  |
| **DR, n (%)** |  |  |  |  | <0.0001 |
| **No** | 4,995 (79.0) | 3,322 (76.0) § | 2,205 (80.1) | 1,754 (75.4) § |  |
| **Non-advanced** | 774 (12.2) | 595 (13.6) | 300 (10.9) † | 288 (12.4) |  |
| **Advanced** | 554 (8.8) | 455 (10.4) | 248 (9.0) | 283 (12.2) ‡ |  |
| **Any CVD event, n (%)** | 932 (14.7) | 1,502 (34.4) § | 408 (14.8) | 813 (35.0) § | <0.0001 |
| **Any coronary event, n (%)** | 476 (7.5) | 1,125 (25.7) § | 219 (8.0) | 595 (25.6) § | <0.0001 |
| **AMI, n (%)** | 336 (5.3) | 813 (18.6) § | 151 (5.5) | 458 (19.7) § | <0.0001 |
| **Any cerebrovascular event, n (%)** | 365 (5.8) | 499 (11.4) § | 150 (5.4) | 291 (12.5) § | <0.0001 |
| **Stroke, n (%)** | 162 (2.6) | 176 (4.0) § | 60 (2.2) | 117 (5.0) § | <0.0001 |
| **Any peripheral event, n (%)** | 263 (4.2) | 311 (7.1) § | 128 (4.6) | 193 (8.3) § | <0.0001 |
| **Ulceration/gangrene, n (%)** | 205 (3.2) | 150 (3.4) | 87 (3.2) | 90 (3.9) | 0.470 |

Values are n (%). *P* values for comparison among groups using the χ^2^ test, 3 df. Post-hoc multiple comparison using the χ^2^ test, 1df: * *P*<0.0001, † *P* at least <0.005, and ‡ *P* at least <0.05 vs. each other group; § *P*<0.0001, and || *P* at least <0.05 vs. groups with no symbol. RIACE = Renal Insufficiency And Cardiovascular Events; eGFR = estimated glomerular filtration rate; CKD = chronic kidney disease; DR = diabetic retinopathy; CVD = cardiovascular disease; AMI = acute myocardial infarction.
